# A systematic comparison of short-term and long-term mortality prediction in acute myocardial infarction using machine learning models

**DOI:** 10.1186/s12911-025-03052-1

**Published:** 2025-06-05

**Authors:** Yawei Yang, Junjie Tang, Liping Ma, Feng Wu, Xiaoqing Guan

**Affiliations:** 1 Department of Cardiology, Yueyang Hospital of Integrated Traditional Chinese and Western Medicine, Shanghai University of Traditional Chinese Medicine, Shanghai, 200437 China; 2 Institute of Interdisciplinary Integrative Medicine Research, Shanghai University of Traditional Chinese Medicine, Shanghai, 201203 China; 3 Department of Cardiology, Changhai Hospital of Shanghai, Shanghai, 200433 China

**Keywords:** Acute myocardial infarction, Machine learning, Mortality prediction, Risk factors, Explainable machine learning

## Abstract

**Background and objective:**

The machine learning (ML) models for acute myocardial infarction (AMI) are considered to have better predictive ability for mortality compared to conventional risk scoring models. However, previous ML prediction models have mostly been short-term (1 year or less) models. Here, we established ML models for long-term prediction of AMI mortality (5 years or 10 years) and systematically compare the predictive capabilities of short-term models versus long-term models across varying survival time periods.

**Methods:**

An observational retrospective study was conducted to analyse mortality prediction in patients with varying survival times. A total of 4,173 patients were enrolled from two different hospitals in China. The dataset was allocated into three groups and an external test set based on their survival duration: the 1-year group (*n* = 3,626), the 5-year group (*n* = 2,102), the 10-year group (*n* = 721), and the external test set (*n* = 545). A comprehensive set of 53 variables was collected and utilized for model development. Mortality prediction was analysed using oversampling and feature selection methods coupled with machine learning algorithms. SHapley Additive exPlanations (SHAP) values were utilized to quantify the feature importance of AMI risk. The best-performing models from each group were selected for a systematic comparison of predictive accuracy using the external test set with follow-up exceeding 10 years but with varying survival times.

**Results:**

For the 1-year model, the RF model achieved the best performance on the test dataset, with an F1 score of 97.81% using only oversampling without feature selection. Conversely, in the case of the 5-years, the combination of LASSO and RF yielded the best performance, achieving F1 scores of 91.35% with both feature selection and oversampling. The best model of 10-years group was SVM with only oversampling without feature selection, yielding an F1 score of 80.7%. Age, BNP, and the Killip classification of AMI were consistently identified as robust predictors across all three groups. This underscores aging as a critical AMI risk factor contributing to mortality. However, despite the model’s success, when examining the actual survival times of the 545 patients, of which 64% survived beyond 5 years and 37% beyond 10 years, the 1-year model failed to distinguish between these patients, predicting all as low risk. This highlights the limitation of short-term models, indicating their inability to accurately predict actual long-term survival times despite being commonly used in AMI mortality prediction.

**Conclusions:**

The study identifies Age, BNP, and Killip classification as consistent predictors of AMI mortality across all groups, with Age being the most significant factor. CBC parameters and renal biomarkers were pivotal in short-term models, while therapeutic interventions gained prominence over time. The 10-year group emphasised disease severity and treatment history, indicating survivorship bias. Short-term models, typically relying on data spanning 1 year or less, commonly established as predictive models for AMI risk, demonstrate limited capability in accurately predicting actual long-term survival times. To effectively issue early warnings for genuine long-term mortality risks, it is imperative to collect longer-term patient information and establish ML prediction models tailored to long-term outcomes. Further research is warranted to validate these findings in diverse populations.

**Supplementary Information:**

The online version contains supplementary material available at 10.1186/s12911-025-03052-1.

## Introduction

Cardiovascular disease (CVD) remains a leading cause of global mortality and a significant global health issue [1–3]. Among them, acute myocardial infarction (AMI) stands out as one of the most lethal CVDs, which is caused by a sudden blockage of blood flow to the heart, typically due to the rupture of an unstable plaque and subsequent thrombus formation. Accurate individual risk prediction for AMI in clinical settings is highly desirable to guide clinicians in rapid clinical decision-making and choosing appropriate treatment strategies, support screening of high-risk individuals, and aid in patient risk stratification [4].

AMI risk is influenced by various factors, such as age, gender, comorbidities, and high heart rate. Over the years, several risk scoring methods have been proposed to predict short-term and long-term mortality rates in patients with AMI, including the GRACE, TIMI, and Framingham Risk Score [5–9]. These tools predominantly draw upon conventional statistical approaches, such as the Cox proportional hazards model and logistic regression, to estimate risk [10, 11]. Furthermore, most of the conventional approaches were designed based on average features [5–11]. However, each individual AMI patient presents with different aetiologies, symptoms, and signs. Thus, the guidelines may not be applicable to some patients, making it challenging to accurately identify individual risk or longitudinally monitor the risk of AMI and conduct risk-stratified management. Therefore, the development of new models capable of automatically identifying, longitudinally monitoring, and timely alerting each patient’s risk of myocardial infarction holds practical significance.

In recent years, with the development of machine learning (ML) technology, researchers have started to explore the application of ML methods in cardiovascular disease risk prediction. ML methodologies offer distinct advantages over traditional statistical approaches, with the capacity to process larger datasets, identify complex patterns in the data, and make more accurate predictions [12–14]. For instance, research conducted by Alaa et al. highlighted the superior performance of ML-based models in predicting cardiovascular risk compared to conventional scoring systems [15]. Nishi M et al. proved that their RF-based ML AMI predictor had better prediction performance than conventional scoring systems, including GRACE and TIMI [14]. Additionally, according to Steele et al. [16], the benefits of ML algorithms include a decrease in the time required for feature selection, the ability to interpret non-parametric and non-linear interactions, and the accommodation of missing data through the implementation of multiple imputations. Roudini et al. constructed ML models with 139 AMI patients and 11 features, including two novel biomarkers, to enhance long-term mortality prediction performance [17].

To our knowledge, although numerous ML models have been established to predict AMI mortality rates, the majority of these are short-term models (1 year or less), and there are few existing models to predict long-term mortality rates(5-years or 10-years) [5, 12–15, 18–27]. Furthermore, a systematic comparison of the predictive performance between short-term and long-term models has not been conducted. The objective of this study was to develop long-term prediction models and to systematically compare the predictive capabilities of short-term models versus long-term models across varying survival time periods. Additionally, SHapley Additive exPlanations (SHAP) values were utilized to identify the important factors contributing to the predictions of different models.

## Methods

### Study data

The subjects of this study were selected from patients who were hospitalized in the Cardiovascular Department of Yueyang Hospital of Integrated Traditional Chinese Western Medicine of Shanghai University of Traditional Chinese Medicine and Changhai Hospital of Shanghai between January 1, 2009, and December 31, 2020. These patients were primarily diagnosed with AMI following their discharge, which included both ST-segment Elevation Myocardial Infarction (STEMI) and Non-ST-segment Elevation Myocardial Infarction (NSTEMI). The diagnosis of AMI was based on the definition of AMI in General Definition of Myocardial Infarction, 4th Edition [28], which divides this disease into various types such as type 1, 2, 3, 4a, 4b, 4c, and 5. Among them, type 4a is a myocardial infarction related to percutaneous coronary intervention (PCI), while type 5 is a myocardial infarction caused by coronary artery bypass grafting (CABG). Both of these types belong to myocardial damage caused by surgical operations, but it should be noted that the surgery itself was successful, and the prognosis of the patients was usually good. In this study, the main reason for excluding type 4a and type 5 myocardial infarctions was that the clinical data of such patients might interfere with the effectiveness of the machine learning model, thereby affecting the accuracy of the research results. Patients with AMI type 1, 2, 3, 4b and 4c were included in the study, while patients with AMI type 4a and type 5 were excluded. This study was approved by the Ethics Committee of both hospitals.

### Data collection

Data collection involved a thorough review of the electronic medical record system to gather a comprehensive dataset on each patient. This included personal demographic information (age, gender, duration of hospital stays, height, and weight), medical and family histories, admission examination findings, clinical diagnoses, treatment regimens, complications, in-hospital survival status, and the cause of death. This information was meticulously extracted and organized from the patient’s medical history records. In our study, we conducted a follow-up of over 10 years for 4,173 patients who were admitted to the hospital. The time of death was documented for each patient. In cases where the patient remained alive at the final follow-up, the date of this final contact was noted instead. All deaths were defined as all-cause deaths during hospitalization.

**Fig. 1:**
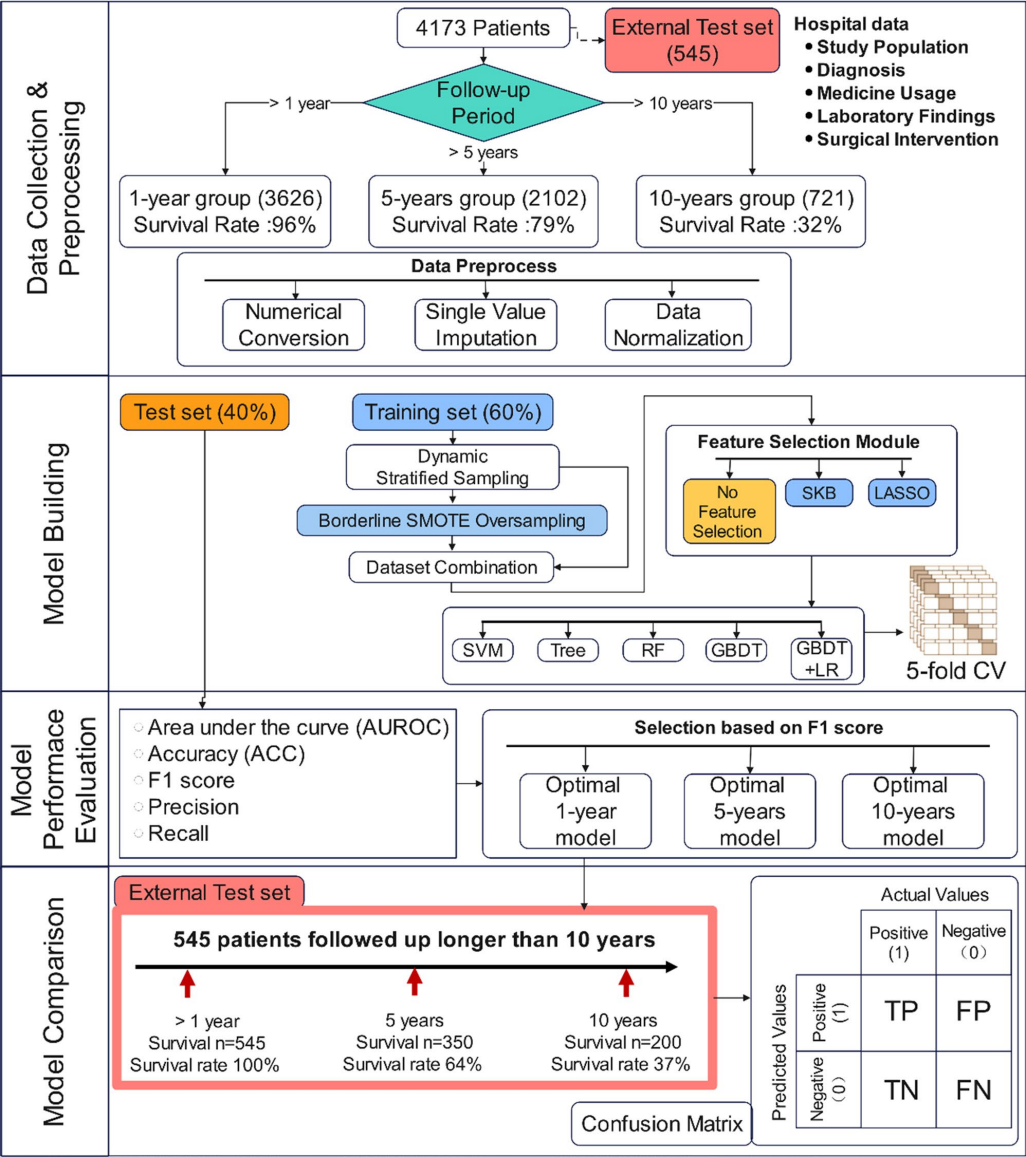
Machine learning model building flowchart

### Data grouping

An independent test set comprising 545 patients was first derived from the complete dataset (total *n* = 4,173). This subset included patients surviving 1–5 years (*n* = 195), 5–10 years (*n* = 150), and over 10 years (*n* = 200), with detailed characteristics provided in Table S2. The remaining 3,628 patients were categorised into three groups based on their survival time and follow-up duration: the 1-year group (*n* = 3,626), the 5-year group (*n* = 2,102), and the 10-year group (*n* = 721), as illustrated in Fig. 1. Within each group, patients who survived for less than the predetermined time point were classified as having a high risk of mortality related to AMI (non-survival, labelled as 0), whereas those who survived beyond the time point were considered to have a low risk (survival, labelled as 1). For instance, in the 1-year group, patients who were followed up and survived less than 1 year were identified as high risk, whereas those who lived longer than 1 year were categorized as low risk. In the 5-years group, patients who were followed up for at least 5 years and died were deemed high risk, while those who survived beyond 5 years were considered low risk; data from patients who were not followed up for the full 5 years were excluded. Similarly, for the 10-year group, patients followed up for at least 10 years and deceased were classified as high risk, and those who survived more than 10 years were labelled as low risk; follow-up data falling short of 10 years were omitted.

### Preprocessing of data

The profile of missing data within the dataset was meticulously analysed (see Figures S1-3 and Table S3) to inform the selection of an appropriate imputation method. Furthermore, Little’s MCAR (Missing Completely At Random) test [29] was conducted to assess whether a systematic pattern underlay the missing values. The test yielded a Chi-Square value of 9094.450 with 3849 degrees of freedom, and a p-value of less than 0.001. Given that the p-value was significant, this indicated the presence of a non-random pattern in the missing data. Consequently, we employed a multiple imputation approach to address this issue [30]. Specifically, we opted for a Linear regression-based multiple imputation method, with survival time incorporated as an analysis weight variable. To ensure robustness, the maximum number of parameters in the imputation model was set to 1000.

Additionally, the impact of dimensional differences between various variables on the training effectiveness of machine learning models was considered. Therefore, all data is processed through numerical conversion and Min-Max normalization before being input into machine learning models [31]. Both methods are commonly used in data cleaning for the construction of machine learning classification models.

### Model development

Three models corresponding to different survival time durations were constructed: the 1-year, 5-years, and 10-year AMI mortality prediction models, each based on their respective data groups. The overall process of the experimental setting was illustrated in Fig. 1.

#### Data splitting

To construct the 1-year, 5-years, and 10-year AMI mortality prediction models, stratified sampling was employed on the corresponding group data to maintain a consistent distribution of data. The data were then divided into training (60%) and test (40%) subsets (Table 1). We then used a 5-fold cross-validation technique on the training set to avoid model overfitting and for hyperparameter tuning.

**Table 1:** Training and test data splitting for model development

Groups	Training set (60%)	Test set (40%)	Total
1 year	2175	1451	3626
5 years	1260	842	2102
10 years	432	289	721

#### Data imbalance processing

Due to the imbalance of the label proportion, we utilized the Borderline SMOTE1, an oversampling method based on Synthetic Minority Oversampling Technique (SMOTE). This method is a refinement of SMOTE and is specifically designed to address the issue of generating an excessive number of overly similar samples. Borderline SMOTE1 focuses on oversampling minority examples that are in close proximity to the decision boundary, thereby ensuring a more balanced representation of the dataset without introducing unnecessary redundancy. This approach has demonstrated improved True Positive (TP) rates and F-values for minority classes when compared to the traditional SMOTE and random oversampling methods, as evidenced by experimental results [32]. Furthermore, to prevent the data distribution resulting from oversampling based on a minority label from deviating significantly from the distribution of the original data, a dynamic stratified sampling algorithm was developed and implemented prior to the oversampling process. This ensured that only the imbalanced data were oversampled to the predetermined target label proportion (TLP). In this study, where TLP was set at 0.4, datasets with a label proportion (LP) below the TLP or above 1-TLP were partially oversampled using the Borderline-SMOTE1 algorithm. More precisely, the absolute percentage of (TLP-LP) from the training set was stratified-sampled for oversampling. The oversampled portion was then combined with the remaining training set to construct the processed training set, which had an LP equal to the TLP.

#### Feature selection

Feature selection was found to be crucial for optimizing model performance by identifying the most relevant variables and eliminating noise, thereby reducing overfitting and improving model interpretability [33]. In this study, two feature selection methods from scikit-learn were employed: SelectKBest (SKB) and Least Absolute Shrinkage and Selection Operator (LASSO) [34, 35].

- SKB

The SKB function from scikit-learn was applied to the feature selection process in this study. It operates by scoring each feature with a given scoring function, and then selecting the k highest-scoring features [34]. This method is useful for creating a subset of the most relevant features that can improve the performance of machine learning models. By applying SKB, the study was able to identify the top k features that contributed the most to the predictive power of the models, thus enhancing their overall performance. In this study, SKB was utilized with the f_classif function provided by scikit-learn, which is based on the ANOVA F-value for classification tasks, allowing for the selection of either the scores or the p-values as output. Due to the significant differences in data distribution among the various datasets, each dataset underwent feature selection via the SKB function twice to determine the optimal k parameter. In the first iteration, SKB was set to select all features (k = k0 = 50) to calculate the number of significant features, k1, with p-values less than 0.01. In the second iteration, SKB selected the top k1 features based on their scores, thus completing the feature selection process.

- LASSO

The LASSO was applied for feature selection in this study, as it is a linear regression model with L1 regularization that adds a penalty to the absolute size of the coefficients. This encourages sparse solutions, effectively reducing the number of features by driving the coefficients of less important features to zero. The strength of the regularization is controlled by the alpha parameter, which balances the trade-off between the accuracy of the model and the number of selected features. In this study, the alpha parameter was set to 0.0001 to avoid selecting too few features, which could potentially lead to poor performance in subsequent model training. By choosing an appropriate alpha value, the study aimed to retain a sufficient number of relevant features to ensure optimal model performance [35].

#### Predictive models

In this study, several types of models were employed, including Support Vector Machine (SVM), Decision Tree, Random Forest (RF), and Gradient Boosting Decision Tree (GBDT). Additionally, a hybrid model, which combined GBDT with Logistic Regression (LR), was utilized for further optimization.

### Model evaluation

In assessing the predictive performance of models, mainstream evaluation metrics such as AUROC, accuracy, and F1 score were all employed in this study. AUROC, or Area Under the Receiver Operating Characteristic curve, was used as a metric to evaluate the overall performance of binary classification models. It represents the probability that a randomly chosen positive instance is ranked higher than a randomly chosen negative instance, regardless of the threshold used to classify instances as positive or negative. The AUROC value ranges from 0 to 1, with a higher value indicating better model performance [36]. 

The F1 score serves as a metric for gauging the effectiveness of binary classification models in machine learning, integrating both precision and recall metrics. Unlike accuracy or AUROC alone, the F1 score provides a more equitable evaluation of the model’s actual predictive prowess, offering a truer reflection of its ability to accurately predict outcomes. Consequently, the F1 score was utilized to assess the performance of the models aimed at predicting the mortality risk of AMI [37]. 

Additionally, SHAP values were utilized to evaluate the influence of features on the predictions made by the multivariate models, namely the AMI risk factors [38]. To comprehensively assess the impact of all features on the AMI survival prediction made by models, the models subjected to SHAP evaluation were developed without undergoing feature selection.

### External test setting

To compare the predictive capabilities of these three models for long-term mortality (10-year) risk, an additional independent external test set containing 545 patients was created. This set included patients whose true survival time exceeded one year and whose follow-up duration extended beyond 10 years, with survival times distributed as follows: 1–5 years (survival rate 36%, *n* = 195), 5–10 years (survival rate 28%, *n* = 150), and over 10 years (survival rate 37%, *n* = 200) respectively (Fig. 1). Furthermore, the labels for each model were adapted to maintain consistency with the nature of their original tasks, as shown in Fig. 5. For instance, the 5-years model predicted the risk at the 5-years time point, where patients surviving less than 5 years were labelled as high risk (*n* = 195), while those surviving beyond 5 years were classified as low risk (*n* = 350). Similarly, for the 1-year model, patients surviving less than 1 year were labelled as high risk, and for the 10-year model, patients surviving less than 10 years were labelled as high risk, with the respective numbers of high and low-risk patients as per the model’s criteria. It is noteworthy that this dataset did not appear in the training or test datasets of the 1-year, 5-years, or 10-year models, which ensured an unbiased comparison of the three models on this dataset.

## Results

### Patient characteristics

According to the data collected, a total of 4,173 patients were included in the analysis. Of these, 545 patients with follow-up durations exceeding 10 years were designated as the external test set, while the remaining patients were divided into three groups based on survival time to train predictive models: the 1-year, 5-year, and 10-year groups. In terms of population (and mortality), the numbers of 1-year, 5-years, and 10-years group were 3626 (3.9%), 2102 (21.1%), and 721 (68.3%). Furthermore, the statistical analysis was accomplished based on the groups individually (Table S1).

Among the three groups, there was a mutual significant difference between survivors to non-survivors in terms of 6 features from 3 different categories (*p* < 0.0001). Majority of them were 4 diagnosis features (Killip classification of AMI, lesion range, operation time, lesion severity). In comparison, only 1 study population feature (gender) and 1 surgical intervention feature (revascularization) shared common significance in all groups.

Generally speaking, the long-term study groups had more significant features than the shorter ones, as the total number of 10-years group was the maximum (26), followed by the 5-years (13) and the 1-year group (10). The four categories of laboratory findings, diagnosis, surgical intervention, and medicine usage exhibited varying degrees of increment. Especially in the case of laboratory findings, the number increased from 2 in the 1-year group to 7 in the 10-year group, suggesting a potential connection to disease progression and physiological changes. The overall increasement in multiple variables across these different categories highlights the necessity for a comprehensive patient assessment during long-term observations. In contrast, study population showed the least sensitivity to changes over time, with consistent significant features between the 5-years and 10-year groups.

### ML prediction performance

Due to the significant impact of data imbalance on model performance, where the proportion of deaths accounted for only a minority in the 1-year and 5-years data, with survival rates as high as 96% and 79% respectively, direct use of machine learning training might result in poor performance and a tendency for positive class overfitting. To address this issue, the Borderline SMOTE1 algorithm was utilized for oversampling. The prediction performance with and without oversampling was first compared across the three groups of models.

As shown in Table 2, the performance of oversampled models was evaluated based on the AUROC, and it was observed that in the 1-year and 5-years groups, the majority of models exhibited improved performance after oversampling compared to before. As for the 10-year data, since the label proportions in the dataset were relatively balanced, both non-oversampled and oversampled models performed well, resulting in little difference in model performance due to oversampling. To access whether the improvement in the AUROC of models, the DeLong’s tests [39] were performed to determine whether the changes were statistically significant (Table S4). Generally, the adoption of oversampling techniques for data augmentation was found to be potentially beneficial in enhancing the prediction performance of machine learning models trained on imbalanced data.

**Table 2:** The AUROC of ML models with and without oversampling based on a 40% test set

	1 year				
	SVM	Tree	RF	GBDT	GBDT + LR
No Oversampling	0.7085	**0.6852**	0.8006	0.7991	0.6934
Borderline SMOTE1	**0.7595**	0.6042	**0.8238**	**0.8164**	**0.7272**
	**5 years**				
	SVM	Tree	RF	GBDT	GBDT + LR
No Oversampling	**0.7952**	0.6187	**0.8070**	0.8209	0.7577
Borderline SMOTE1	0.7178	**0.6551**	0.7622	**0.8273**	**0.7875**
	**10 years**				
	SVM	Tree	RF	GBDT	GBDT + LR
No Oversampling	0.9238	**0.8237**	**0.9471**	**0.9533**	0.9508
Borderline SMOTE1	**0.9268**	0.7979	0.9316	0.9449	0.9378

The better AUROC value for each model was highlighted

Feature selection has been demonstrated to enhance model accuracy, interpretability, and efficiency by reducing overfitting and training time through the selection of the most relevant features. In this study, two common oversampling techniques, SKB and LASSO, were employed. The detailed performance evaluation of the best ML model predicting AMI risk was presented in Table 3, Figure S4, and Table S5-S7. The F1 score, being the harmonic mean of precision and recall, serves as a metric that balances the two measures to provide a composite assessment of a model’s performance. In other words, a high F1 score indicates that the model not only considers true positives (TP) but also considers both false positives (FP) and false negatives (FN) in its calculations, which is often desirable in many real-world applications. Hence, the F1 score was used to compare the impact of feature selection on the same base ML model. As seen in Table 3, the F1 scores of almost all models were improved to some extent, indicating the effectiveness of feature selection methods in data-driven ML model construction for predicting AMI risk.

**Table 3:** The F1 score of ML models with and without feature selection based on a 40% test set

	F1 score		
	No Feature Selection	SKB	LASSO
**1 year**			
SVM	0.9610	**0.9756**	0.9614
Tree	**0.9662**	0.9536	0.9501
RF	**0.9781**	**0.9781**	0.9774
GBDT	0.9769	**0.9774**	0.9758
GBDT + LR	0.9769	**0.9774**	0.9758
**5 years**			
SVM	0.8754	**0.9067**	0.9034
Tree	0.8764	0.8756	**0.8849**
RF	0.8947	0.9086	**0.9135**
GBDT	0.9095	**0.9118**	0.9067
GBDT + LR	0.9095	**0.9118**	0.9067
**10 years**			
SVM	**0.8075**	**0.8075**	0.7582
Tree	0.7073	**0.7101**	**0.7101**
RF	0.7671	**0.7867**	0.7639
GBDT	**0.7947**	0.7517	0.7324
GBDT + LR	**0.7947**	0.7517	0.7324

The best F1 score of the feature selection methods for each model was highlighted.【粗体, 星号】

In summary, the performance of RF, GBDT, and GBDT + LR ranked among the top three in both 1-year and 5-years groups, whereas SVM topped in the 10-years group, with F1 scores, AUROCs, and accuracy values consistently exceeding 0.75 (Figure S4 and Table S5-S7). The F1 score was chosen as the criterion for selecting the best model. Thus, RF was identified as the optimal model for the 1-year, LASSO + RF for the 5-years, and SVM for the 10-years. These models were employed in subsequent studies, and further detail about their hyperparameters and feature selection were presented in Table S8-S10.

### Feature importance

To understand how variables impact the model’s output on each experiment proposed, we utilized SHAP values to quantify the significance of features in the predictive models for 1-year, 5-years, and 10-years groups. SHAP provides a unified measure of feature importance that allocates the contribution of each feature to the prediction for each individual instance in the dataset to evaluate the AMI risk factors.

**Fig. 2:**
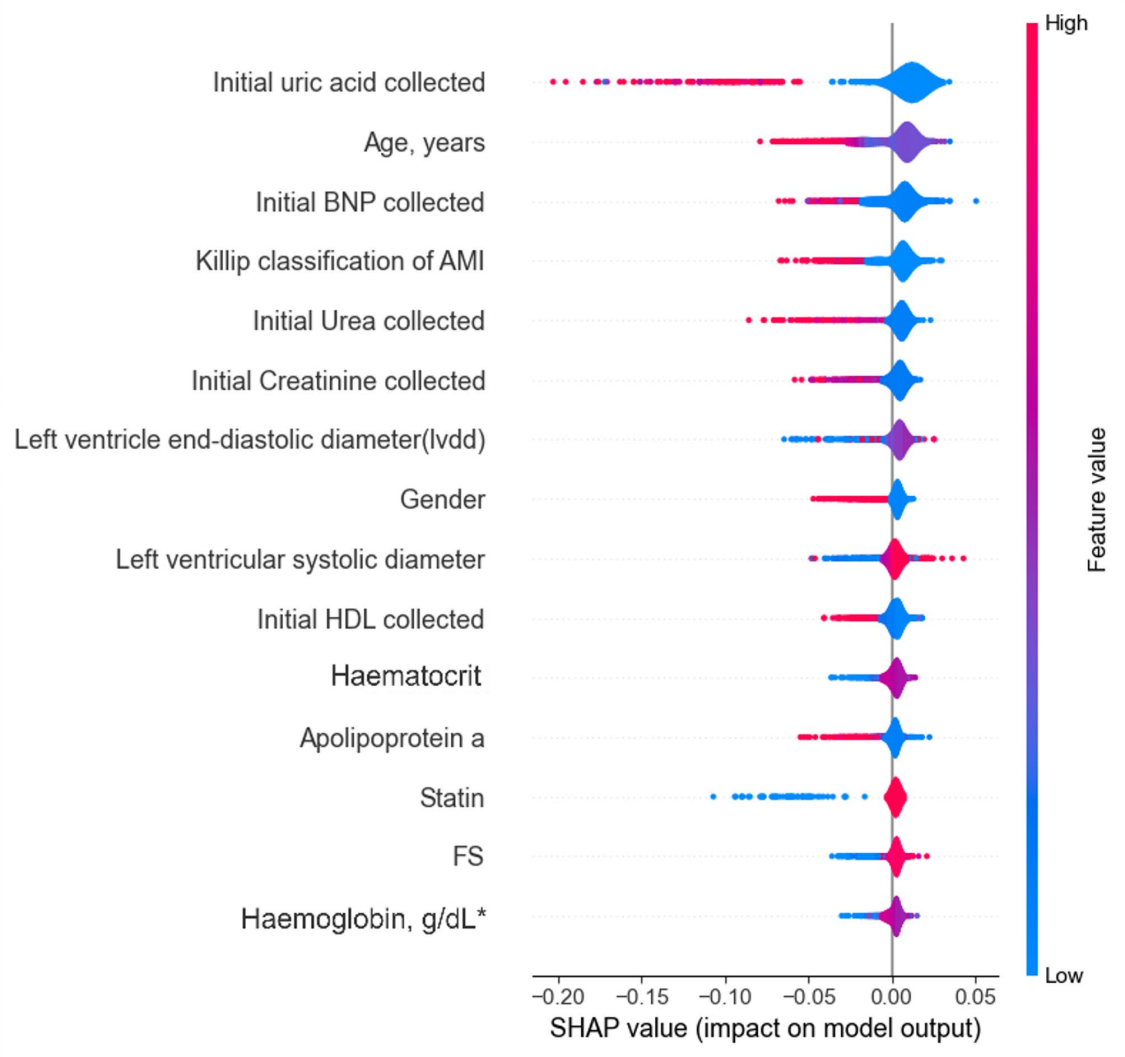
Feature impact estimation of the 1-year RF model on AMI mortality risk

**Fig. 3:**
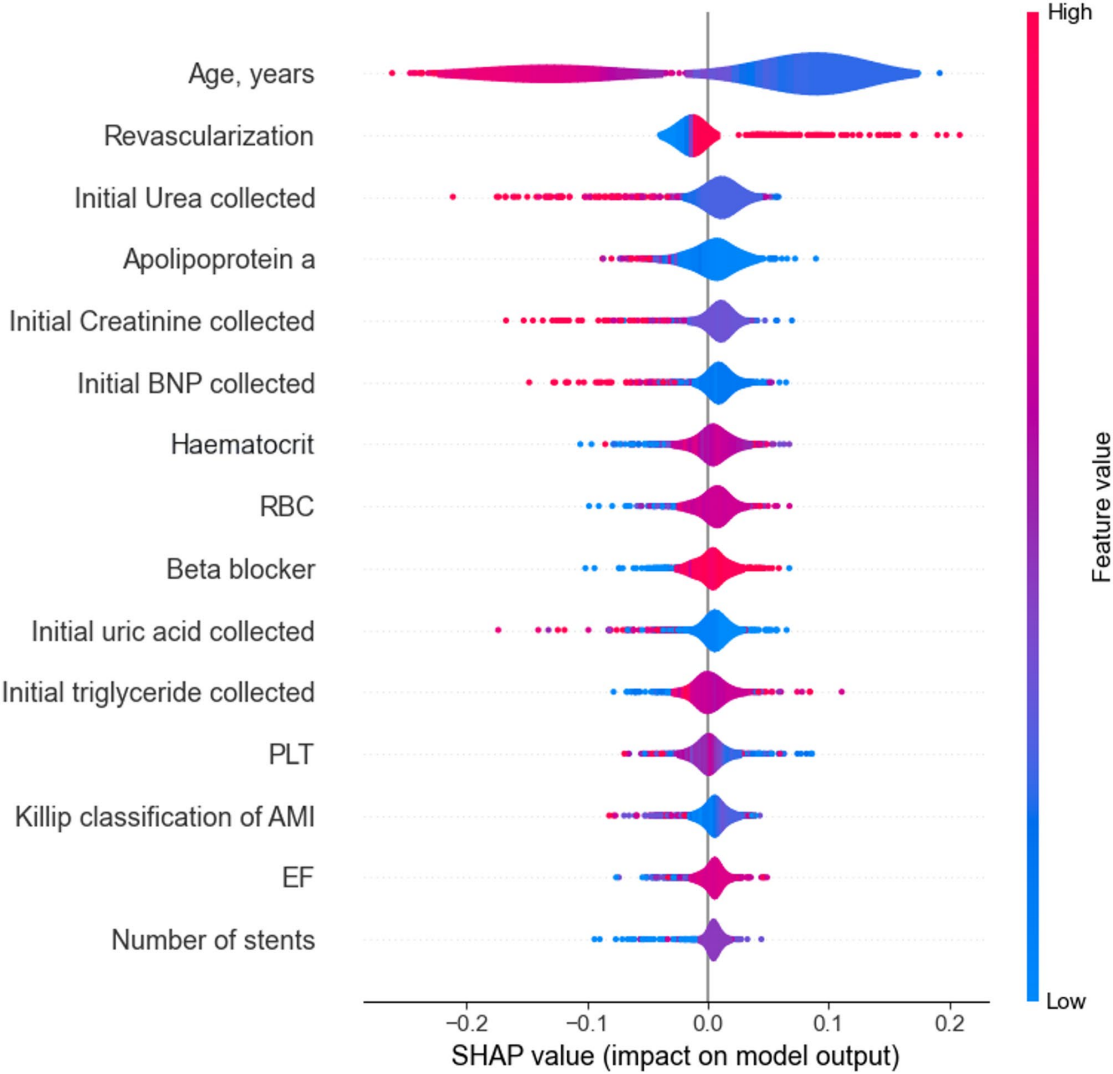
Feature impact estimation of the 5-years RF model on AMI mortality risk

**Fig. 4:**
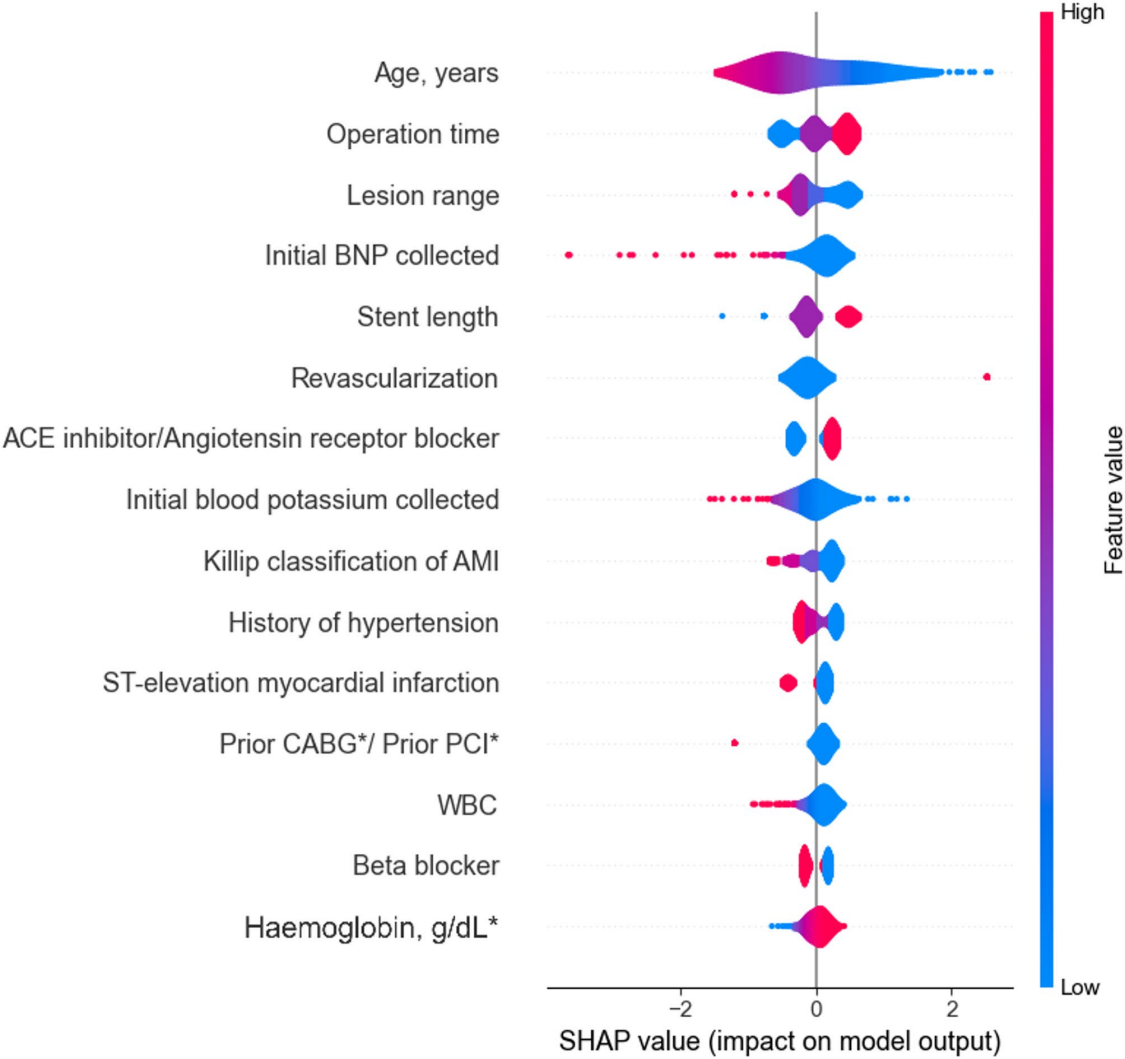
Feature impact estimation of the 10-years SVM model on AMI mortality risk

As is shown in Figs. 2, 3 and 4, the top 15 AMI risk factors of each group were measured and depicted. Each dot on the plot represents a SHAP value for a feature and an instance. The position on the y-axis is determined by the feature, and on the x-axis by the SHAP value. The colour represents the value of the feature from low to high. The features are ordered by the sum of absolute SHAP values over all instances, with the most important feature at the top.

Notably, 3 out of the top 15 features — namely Age, BNP, and the Killip classification of AMI — were robustly identified as consistently important across all three groups. This underscores their significant impact on AMI mortality throughout the study population, irrespective of the survival timeframe under examination. Particularly, age emerged as the most significant feature in all three groups, highlighting aging as one of the most crucial AMI risk factors contributing to mortality across all groups. Additionally, all three groups highlighted at least two Complete Blood Count (CBC) parameters (e.g. Haematocrit, Haemoglobin, RBC and WBC) as one of the pivotal AMI risk factors. This finding reinforces the diagnostic value of CBC-derived biomarkers in assessing prognosis and guiding clinical decision-making for AMI patients.

In the 1-year and 5-year models, the results identified kidney function-related parameters (uric acid, urea, and creatinine) as significant determinants of AMI risk, implicating a potential association between renal function and AMI outcomes. Notably, gender emerged as a critical risk factor in the 1-year group but was absent from the top 15 predictors in the longer-term cohorts (5-year and 10-year groups). While initial laboratory findings exerted a consistent influence on risk prediction in the shorter-term groups, therapeutic interventions demonstrated escalating importance over time. Specifically, revascularization and β-blocker use exhibited pronounced effects on predicted AMI risk in the 5-year and 10-year groups, as evidenced by distinct patterns in the distribution of data points in Figs. 3 and 4.

In the 10-year group, the majority of risk factors were linked to disease severity and therapeutic history rather than baseline laboratory metrics (Fig. 4). This shift may reflect survivorship bias and the waning relevance of initial biomarkers over extended follow-up periods. The diminishing significance of early laboratory findings could be attributed to competing risks, evolving disease trajectories, or the cumulative impact of treatments such as revascularization and β-blockers. Further research is warranted to explore how therapeutic interventions modulate long-term AMI risk and to validate these findings in diverse cohorts.

### Evaluation of 1-year, 5-years and 10-years model performance across varying patient survival times

**Fig. 5:**
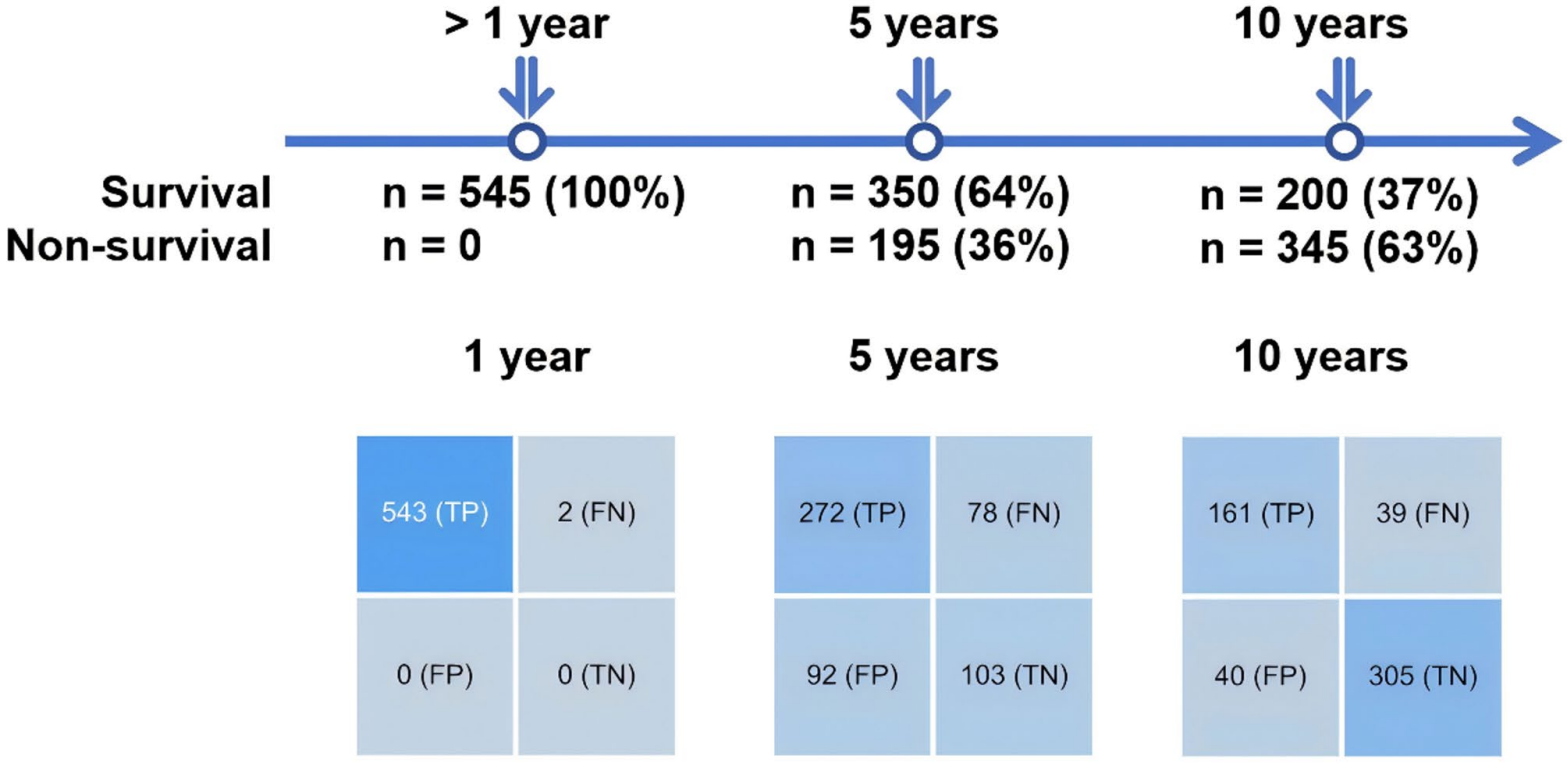
Evaluation of 1-year, 5-years and 10-years model performance across varying patient survival time

In order to evaluate the performance of the best ML models for the 1-year, 5-years, and 10-years groups in predicting the risk of AMI-related mortality across varying patient survival times, an independent external test set was collected. This dataset comprised 545 patients who had been followed up for over 10 years, with their survival times exceeding 1 year (Fig. 5).

The AMI mortality risk for the 545 patients was predicted using the top models from each of the three groups. To ensure an unbiased comparison, the patient labels were adjusted according to the corresponding model’s time frame. For instance, for the 1-year model, all 545 labels were considered as 1. For the 5-years model, 350 labels were considered as 1, and 195 as 0. Similarly, for the 10-year model, 200 labels were considered as 1, and 345 as 0. We employed the model corresponding to each time frame to predict the current mortality rate. The prediction results were presented in a confusion matrix as shown in Fig. 5. In the context of a confusion matrix, a lower ratio of FP (high-risk instances predicted as low-risk) and FN (low-risk instances predicted as high-risk) indicates better prediction performance by the model. The results indicated that the 1-year model successfully predicted survival times exceeding 1 year for most of the 545 data points (with all predictions being TP, and no FP or FN), suggesting that all instances were predicted as low-risk. For the 5-years and 10-year models, the F1 scores were 0.7619 and 0.8030, respectively, indicating that all three models exhibited good predictive performance on the external validation set.

However, when considering the actual survival times of the 545 patients, 64% of them actually survived beyond 5 years, and 37% survived beyond 10 years. Unfortunately, the 1-year model was unable to distinguish between them and predicted all patients as low risk. This outcome suggests that short-term models relying on data from short time data (1 year or less), which are commonly established as predictive models for AMI mortality, lack the capability to accurately predict actual long-term survival times. Therefore, it is imperative to collect longer-term patient information and establish long-term ML prediction models to effectively issue early warnings for real long-term mortality risks.

## Discussion

In this study, we conducted a 10-year follow-up of 4,173 patients. Based on the actual follow-up data, we developed short-term (1-year), medium-term (5-years), and long-term (10-year) AMI mortality prediction models. For the one-year model, the RF model achieved the best performance on the test dataset, with an F1 score of 98.07%, using only oversampling without feature selection. However, for the 5-years and 10-year models, the combination of LASSO and RF (LASSO + RF) demonstrated the best performance, with F1 scores of 89.57% and 83.44%, respectively, utilizing both feature selection and oversampling.

Short-term models, such as those with a time horizon of 1 year or less, currently represent the mainstream in acute myocardial infarction (AMI) prediction [12–14, 18, 19, 21–25]. Recently, Roudini et al. proposed a long-term AMI prediction model focusing on 14-year all-cause mortality based on a cohort of 139 AMI patients [17]. In this study, we developed time-specific models (1-, 5-, and 10-year) using long-term follow-up data from 4,173 AMI patients. Furthermore, to explore whether the prediction risks of short-term models are related to the actual survival time, we conducted performance evaluations on an external test dataset consisting of 545 patients who had been followed up for more than 10 years with varying survival times ranging from 1 year to over 10 years. However, as shown in Fig. 5, the uniform prediction of low risk by the 1-year model underscored the limitation of short-term models in predicting long-term survival. This outcome suggests that when establishing or utilizing ML prediction models, careful consideration should be given to the applicability of the models. Importantly, the findings also emphasize the imperative of collecting long-term mortality information to develop predictive models if accurate predictions of long-term survival times are required.

SHAP values identified age, BNP levels and Killip classification as consistent predictors of AMI mortality across all cohorts, with age being the strongest determinant. CBC parameters were also highlighted as key biomarkers. Renal function parameters were found to significantly impact short-to-medium term risk, while gender emerged as a critical factor solely in the short term. reinforced our findings These findings are consistent with previously reported AMI risk factors and the SHAP results from recent short-term studies [26, 27]. Yang et al. and Shakhgeldyan et al. both studied in-hospital mortality using SHAP values and highlighted the importance of age, Killip classification and creatinine as critical predictors of AMI mortality [26, 27]. Moreover, Yang et al. also reported results similar to ours regarding the significance of CBC and renal function parameters [27]. Although the underlying mechanism linking aging to AMI mortality remains unclear, there is a growing body of research focusing on the relationship between cardiovascular aging and heart failure. For instance, according to Chen et al., Klotho insufficiency accelerates cardiac aging by compromising the Nrf2-GR pathway [40]. Similarly, Sano et al. found that the occurrence of mosaic loss of Y chromosome (mLOY) within the hematopoietic system is linked to an increased risk for age-related cardiovascular disease, as well as the mortality resulting from heart failure [41]. Additionally, serum urea nitrogen, creatinine, uric acid and B-type natriuretic peptide have been proved as strong AMI risk factors by numerous articles and included into standard clinical guidelines over the past decades [4, 5, 42–47].

In our study, SHAP values also revealed that therapeutic interventions, such as revascularisation and β-blocker use, increase in importance over extended periods. Within the 10-year group, risk factors shift towards disease severity and treatment history. This may be due to survivorship bias and the diminishing relevance of initial biomarkers. This suggests that the significance of early laboratory findings may decrease over time due to competing risks, disease progression, or treatment effects. Although Roudini et al. used the Gini method to investigate feature importances in their long-term study, they did not include sufficient therapeutic intervention factors or early laboratory findings to confirm our findings [17]. Further research is needed to explore how treatments modulate long-term AMI risk and to validate these findings in diverse populations.

In terms of therapies, revascularization was one of the most effective methods increasing the possibility of post-AMI long term survival over the years. Based on the research conducted by Bangalore et al., there was no significant difference in the risk of mortality between patients undergoing percutaneous coronary intervention (PCI) with everolimus-eluting stents and those undergoing coronary artery bypass grafting (CABG) [48]. Additionally, Rocha et al. discovered that total arterial revascularization was linked to enhanced long-term freedom from major adverse cardiac and cerebrovascular events, mortality, and myocardial infarction. This procedure may be the preferred option for patients with a reasonable life expectancy who require CABG [49].

However, this study is not without its limitations. One notable limitation is its two-centre design, which may restrict the generalizability of the findings to a broader patient population. Additionally, the study only examined indicators at the time of admission, omitting the assessment of changes in these indicators over time. This limitation precludes the ability to measure the trends in indicator variability associated with different durations of survival. Future research should aim to overcome these limitations by incorporating multi-centre designs and longitudinal data collection to capture the dynamic changes in biomarkers and other relevant indicators throughout the follow-up period. In addition, external validation across diverse institutions and patient populations will be undertaken in future work to further assess and enhance the robustness and generalizability of the proposed models.

## Conclusions

In conclusion, this study pioneered the establishment of machine learning prediction models for AMI morality of 1 year, 5 years, and 10 years. Through a systematic comparison of predictive accuracy across short-term and long-term models using a dataset of 545 patients with follow-up exceeding 10 years but varying survival times, it was observed that short-term machine learning models were inadequate in predicting long-term survival times. This underscores the necessity of gathering long-term follow-up information to enhance the accuracy of long-term survival risk predictions. Additionally, age was determined to be the most pivotal factor influencing AMI survival outcomes through the employment of SHAP analysis.

## Electronic supplementary material

Below is the link to the electronic supplementary material.

Supplementary Material 1

Supplementary Material 2

Supplementary Material 3

## Data Availability

The datasets used and/or analysed during the current study available from the corresponding author on reasonable request.
