# Supplementary material for: A systematic comparison of short-term and long-term mortality prediction in acute myocardial infarction using machine learning models
Source: BMC Med Inform Decis Mak. 2025 Jun 5;25:208. doi: 10.1186/s12911-025-03052-1 (PMC12143097; doi:10.1186/s12911-025-03052-1)
Supplement: Supplementary file 1 — Supplementary Material 1 [file 12911_2025_3052_MOESM1_ESM.docx]

## Supplementary Data

**A Systematic Comparison of Short-Term and Long-Term Mortality Prediction in Acute Myocardial Infarction using Machine Learning Models**

Yawei Yang^1^, Junjie Tang^2^, Liping Ma^3^, Feng Wu^1^*, Xiaoqing Guan^2^*

^1^ Department of Cardiology, Yueyang Hospital of Integrated Traditional Chinese and Western Medicine, Shanghai University of Traditional Chinese medicine, Shanghai 200437, China.

^2^ Institute of Interdisciplinary Integrative Medicine Research, Shanghai University of Traditional Chinese Medicine, Shanghai 201203, China.

^3^ Department of Cardiology, Changhai Hospital of Shanghai, Shanghai 200433, China.

## CONTENTS

**Table S1.** Patients’ characteristics for the 1-year, 5-years, and 10-years dataset.

**Table S2.** Patients’ characteristics for the external test set (545 patients).

**Table S3.** Variable summary of the missing values in the complete dataset.

**Table S4.** DeLong’s tests comparing the AUROC of ML models with and without oversampling based on a 40% test set.

**Table S5.** Additional performance metrics based on a 40% test dataset the 1-year ML models with and without feature selection.

**Table S6.** Additional performance metrics based on a 40% test dataset the 5-year ML models with and without feature selection.

**Table S7.** Additional performance metrics based on a 40% test dataset the 10-year ML models with and without feature selection.

**Table S8.** Hyperparameters for Random Forests the best models of 1-year (RF) and 5-years (LASSO + RF) group.

**Table S9.** Hyperparameters for Random Forests the best model of 10-years group (SVM).

**Table S10.** Selected features after the feature selection process for the best model of 5-years group. (LASSO + RF)

**Figure S1.** Overall summary of the missing values in the complete dataset.

**Figure S2.** Heat map for missing value patterns in the complete dataset.

**Figure S3**. Histogram for missing value pattern frequencies in the complete dataset.

**Figure S4.** The receiver operating characteristics (ROC) curves of ML models with or without feature selection based on a 40% test dataset.

**Table S3.** Variable summary of the missing values in the complete dataset.

|  | **Missing** | | **Valid N** | **Mean** | **Std. Deviation** |
| --- | --- | --- | --- | --- | --- |
|  | **N** | **Percent** |  |  |  |
| Stent inner diameter | 963 | 23.1% | 3210 |  |  |
| Stent length | 960 | 23.0% | 3213 |  |  |
| Left ventricle end-diastolic diameter(lvdd) | 954 | 22.9% | 3219 | 8.82 | 12.771 |
| Left ventricular systolic diameter | 953 | 22.8% | 3220 | 5.93 | 8.499 |
| FS | 947 | 22.7% | 3226 | 31.71 | 5.552 |
| EF | 942 | 22.6% | 3231 | 59.18 | 8.802 |
| Apolipoprotein b | 723 | 17.3% | 3450 | 0.96 | 1.945 |
| Initial BNP collected | 622 | 14.9% | 3551 | 400.05 | 676.850 |
| Number of stents | 468 | 11.2% | 3705 |  |  |
| Lesion severity | 463 | 11.1% | 3710 |  |  |
| Lesion range | 462 | 11.1% | 3711 |  |  |
| Gensini score | 455 | 10.9% | 3718 | 55.23 | 35.206 |
| Maximum number of variables shown: 25  Minimum percentage of missing values for variable to be included: 10.0% | | | | | |
| Little's MCAR test: Chi-Square = 9094.450, DF = 3849, p < 0.001 | | | | | |

**Table S4.** DeLong’s tests comparing the AUROC of ML models with and without oversampling based on a 40% test set.

|  | **1-year** | | **5-years** | | **10-years** | |
| --- | --- | --- | --- | --- | --- | --- |
| **Model** | **DeLong Z** | **DeLong P-value** | **DeLong Z** | **DeLong P-value** | **DeLong Z** | **DeLong P-value** |
| SVM | -1.3191 | 0.1871 | -3.7797 | 0.0002 | -0.7712 | 0.4406 |
| Tree | -1.7326 | 0.0832 | -1.5088 | 0.1314 | -1.9446 | 0.0518 |
| RF | -1.2558 | 0.2092 | -2.7955 | 0.0052 | -2.9461 | 0.0032 |
| GBDT | -0.9742 | 0.3299 | -0.8364 | 0.4029 | -1.5248 | 0.1273 |
| GBDT+LR | -0.9532 | 0.3405 | -1.8936 | 0.0583 | -1.6158 | 0.1061 |

**Table S5.** Additional performance metrics based on a 40% test dataset the 1-year ML models with and without feature selection.

| **Methods** | **AUROC** | **Accuracy** | **Precision** | **Recall** | **F1 score** |
| --- | --- | --- | --- | --- | --- |
| SVM | 0.7595 | 0.9256 | 0.9617 | 0.9603 | 0.9610 |
| Tree | 0.6042 | 0.9352 | 0.9634 | 0.9690 | 0.9662 |
| RF | 0.8238 | **0.9573** | 0.9572 | **1.0000** | **0.9781** |
| GBDT | 0.8164 | 0.9552 | 0.9616 | 0.9928 | 0.9769 |
| GBDT+LR | 0.7272 | 0.9552 | 0.9616 | 0.9928 | 0.9769 |
| SKB+SVM | 0.7415 | 0.9524 | 0.9551 | 0.9971 | 0.9756 |
| SKB+Tree | 0.7018 | 0.9125 | **0.9653** | 0.9423 | 0.9536 |
| SKB+RF | 0.8145 | **0.9573** | 0.9584 | 0.9986 | **0.9781** |
| SKB+GBDT | 0.8209 | 0.9559 | 0.9565 | 0.9993 | 0.9774 |
| SKB+GBDT+LR | **0.8306** | 0.9559 | 0.9565 | 0.9993 | 0.9774 |
| LASSO+SVM | 0.7520 | 0.9263 | 0.9617 | 0.9610 | 0.9614 |
| LASSO+Tree | 0.6276 | 0.9056 | 0.9589 | 0.9416 | 0.9501 |
| LASSO+RF | 0.7931 | 0.9559 | 0.9559 | **1.0000** | 0.9774 |
| LASSO+GBDT | 0.8104 | 0.9531 | 0.9621 | 0.9899 | 0.9758 |
| LASSO+GBDT+LR | 0.7430 | 0.9531 | 0.9621 | 0.9899 | 0.9758 |

The model reached the highest value based on each metrics was highlighted.

**Table S6.** Additional performance metrics based on a 40% test dataset the 5-year ML models with and without feature selection.

| **Methods** | **AUROC** | **Accuracy** | **Precision** | **Recall** | **F1 score** |
| --- | --- | --- | --- | --- | --- |
| SVM | 0.7178 | 0.7943 | 0.8761 | 0.8748 | 0.8754 |
| Tree | 0.6551 | 0.7967 | **0.8808** | 0.8719 | 0.8764 |
| RF | 0.7622 | 0.8228 | 0.8792 | 0.9108 | 0.8947 |
| GBDT | **0.8273** | 0.8430 | 0.8689 | 0.9540 | 0.9095 |
| GBDT+LR | 0.7875 | 0.8430 | 0.8689 | 0.9540 | 0.9095 |
| SKB+SVM | 0.8032 | 0.8323 | 0.8395 | 0.9856 | 0.9067 |
| SKB+Tree | 0.6402 | 0.7943 | 0.8750 | 0.8763 | 0.8756 |
| SKB+RF | 0.8195 | 0.8395 | 0.8581 | 0.9655 | 0.9086 |
| SKB+GBDT | 0.7982 | 0.8430 | 0.8514 | 0.9813 | **0.9118** |
| SKB+GBDT+LR | 0.7854 | 0.8430 | 0.8514 | 0.9813 | **0.9118** |
| LASSO+SVM | 0.7861 | 0.8252 | 0.8317 | **0.9885** | 0.9034 |
| LASSO+Tree | 0.6795 | 0.8038 | 0.8591 | 0.9122 | 0.8849 |
| LASSO+RF | 0.8139 | **0.8466** | 0.8555 | 0.9799 | 0.9135 |
| LASSO+GBDT | 0.8085 | 0.8371 | 0.8605 | 0.9583 | 0.9067 |
| LASSO+GBDT+LR | 0.7752 | 0.8371 | 0.8605 | 0.9583 | 0.9067 |

The model reached the highest value based on each metrics was highlighted.

**Table S7.** Additional performance metrics based on a 40% test dataset the 10-year ML models with and without feature selection.

| **Methods** | **AUROC** | **Accuracy** | **Precision** | **Recall** | **F1 score** |
| --- | --- | --- | --- | --- | --- |
| SVM | 0.9268 | **0.8927** | 0.8125 | **0.8025** | **0.8075** |
| Tree | 0.7979 | 0.8339 | 0.6988 | 0.7160 | 0.7073 |
| RF | 0.9316 | 0.8824 | 0.8615 | 0.6914 | 0.7671 |
| GBDT | 0.9449 | **0.8927** | 0.8571 | 0.7407 | 0.7947 |
| GBDT+LR | 0.9378 | **0.8927** | 0.8571 | 0.7407 | 0.7947 |
| SKB+SVM | **0.9457** | **0.8927** | 0.8125 | **0.8025** | **0.8075** |
| SKB+Tree | 0.8031 | 0.8304 | 0.6818 | 0.7407 | 0.7101 |
| SKB+RF | 0.9391 | 0.8893 | 0.8551 | 0.7284 | 0.7867 |
| SKB+GBDT | 0.9437 | 0.8720 | 0.8235 | 0.6914 | 0.7517 |
| SKB+GBDT+LR | 0.9316 | 0.8720 | 0.8235 | 0.6914 | 0.7517 |
| LASSO+SVM | 0.9207 | 0.8720 | 0.8056 | 0.7160 | 0.7582 |
| LASSO+Tree | 0.8031 | 0.8304 | 0.6818 | 0.7407 | 0.7101 |
| LASSO+RF | 0.9352 | 0.8824 | **0.8730** | 0.6790 | 0.7639 |
| LASSO+GBDT | 0.9449 | 0.8685 | 0.8525 | 0.6420 | 0.7324 |
| LASSO+GBDT+LR | 0.9190 | 0.8685 | 0.8525 | 0.6420 | 0.7324 |

The model reached the highest value based on each metrics was highlighted.

**Table S8.** Hyperparameters for Random Forests the best models of 1-year (RF) and 5-years (LASSO + RF) group.

| **Hyperparameter** | **Range** |
| --- | --- |
| 'n_estimators' | range(10,100) |
| 'max_depth' | range(1,20) |
| 'max_features' | ['sqrt', 'log2'] |
| 'criterion' | ['gini', 'entropy'] |
| 'random_state' | [0, 2, 42, 3407] |

**Table S9.** Hyperparameters for Random Forests the best model of 10-years group (SVM).

| **Hyperparameter** | **Range** |
| --- | --- |
| 'C' | (0, 20) |
| 'kernel' | ['linear', 'sigmoid', 'poly', 'rbf'] |
| 'gamma' | (0, 20) |
| 'random_state' | [0, 2, 42, 3407] |

**Table S10.** Selected features after the feature selection process for the best model of 5-years group. (LASSO + RF)

| **Model** | **Feature Names** |
| --- | --- |
| LASSO+RF | Age, years |
|  | ST-elevation myocardial infarction |
|  | Killip classification of AMI |
|  | ST myocardial infarction localization |
|  | History of hypertension |
|  | History of diabetes mellitus |
|  | Current/recent smoker* |
|  | Family history of CHD |
|  | 3 degrees AVB |
|  | Sinus arrest |
|  | Ventricular premature risk classification |
|  | Ventricular vibration |
|  | History of atrial fibrillation* |
|  | In Cardiogenic shock* |
|  | Operation time |
|  | Gensini score |
|  | Prior CABG*/ Prior PCI* |
|  | Lesions in the original stent |
|  | Lesion range |
|  | Lesion severity |
|  | Number of stents |
|  | Stent inner diameter |
|  | Stent length |
|  | Statin |
|  | ACE inhibitor/Angiotensin receptor blocker |
|  | Beta blocker |
|  | WBC |
|  | RBC |
|  | PLT |
|  | Haematocrit |
|  | Haemoglobin, g/dL* |
|  | Initial BNP collected |
|  | Initial Troponin collected |
|  | Initial blood potassium collected |
|  | Initial Urea collected |
|  | Initial Creatinine collected |
|  | Initial uric acid collected |
|  | History of dyslipidemia* |
|  | Apolipoprotein b |
|  | Initial LDL collected |
|  | Initial HDL collected |
|  | Initial triglyceride collected |
|  | Initial total cholesterol collected |
|  | Apolipoprotein a |
|  | Postoperative haemorrhage |
|  | Left ventricle end-diastolic diameter(lvdd) |
|  | Left ventricular systolic diameter |
|  | FS |
|  | EF |
|  | ST AVR elevation |
|  | Revascularization |
| Count | 51 |

**
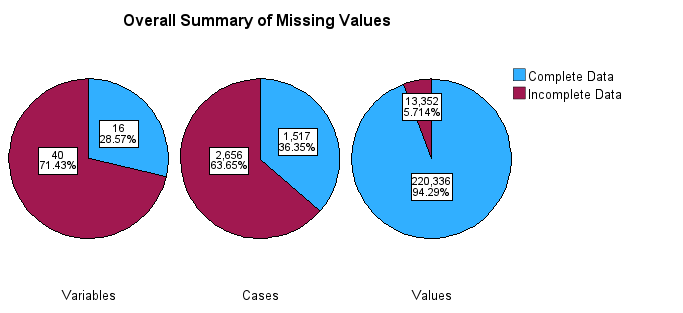
**

**Figure S1.** Overall summary of the missing values in the complete dataset.

**
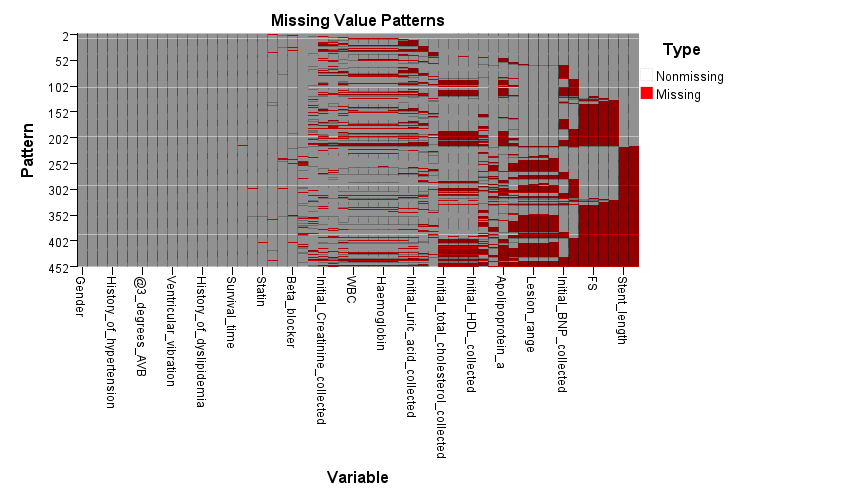
**

**Figure S2.** Heat map for missing value patterns in the complete dataset.

**
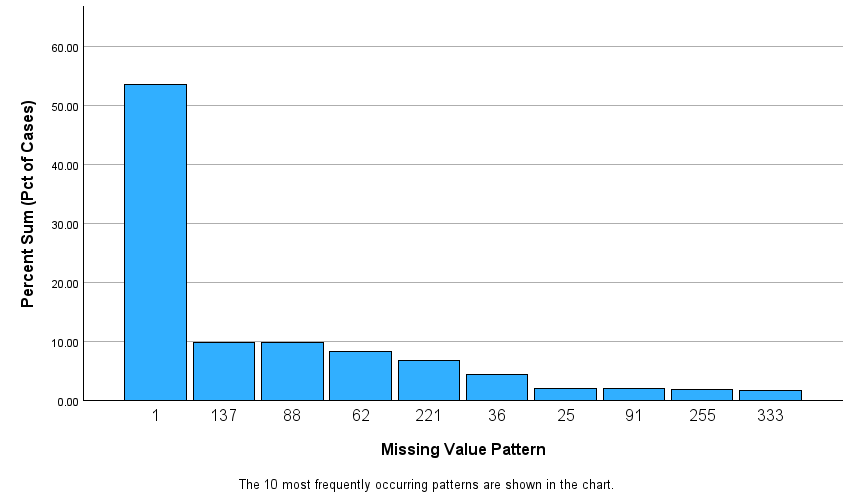
**

**Figure S3.** Histogram for missing value pattern frequencies in the complete dataset.


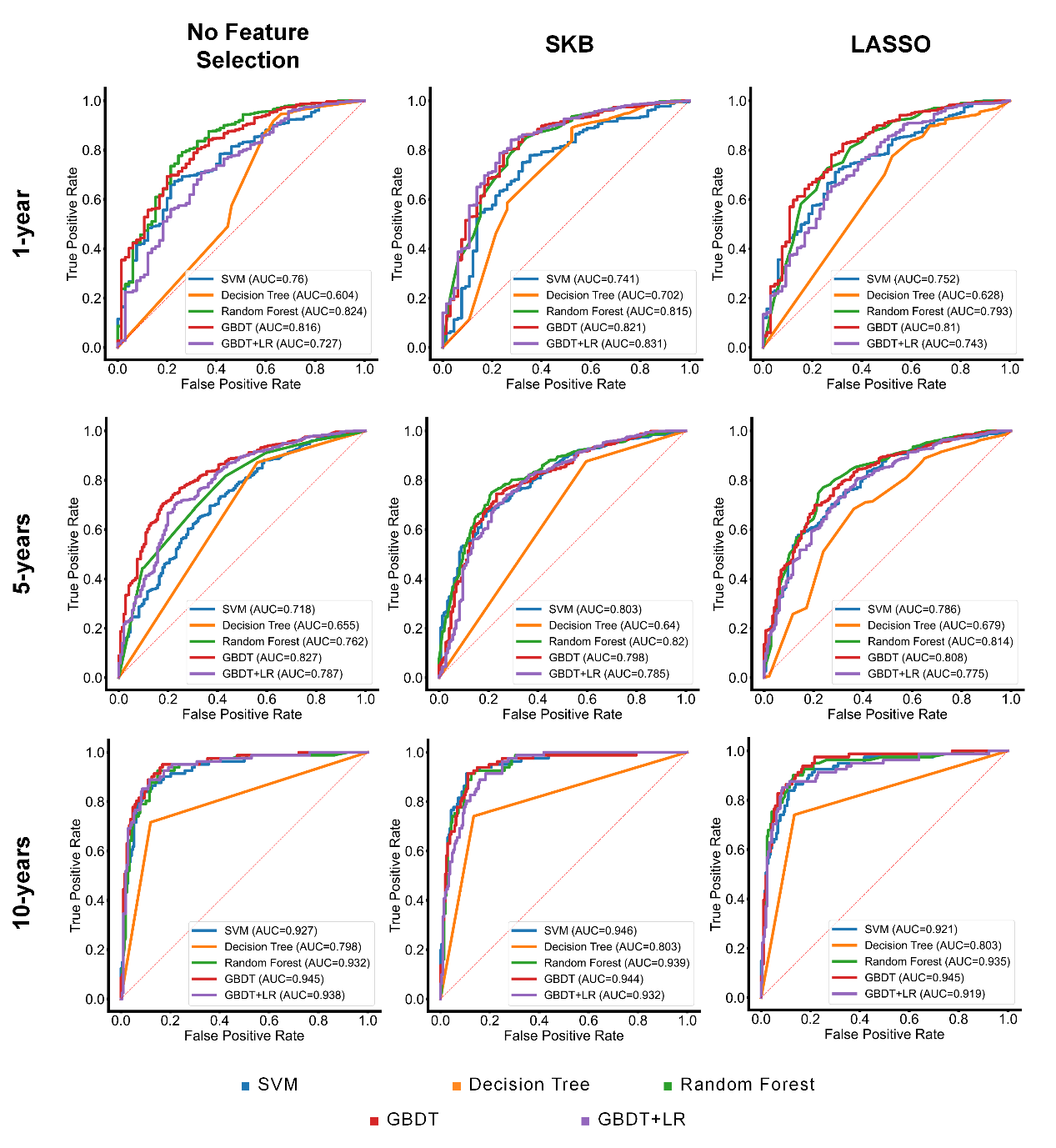


**Figure S4.** The receiver operating characteristics (ROC) curves of ML models with or without feature selection based on a 40% test dataset.
